# Supplementary material for: The effect of intrapartum prolonged oxygen exposure on fetal metabolic status: secondary analysis from a randomized controlled trial
Source: Front Endocrinol (Lausanne). 2023 Jun 27;14:1204956. doi: 10.3389/fendo.2023.1204956 (PMC10335765; doi:10.3389/fendo.2023.1204956)
Supplement: Supplementary file 3 [file DataSheet_3.zip › Result-X101SC21092977-Z01-J001-B1-42/1.MetQuant-QC/Samples_pos-PCA.3D.pdf]

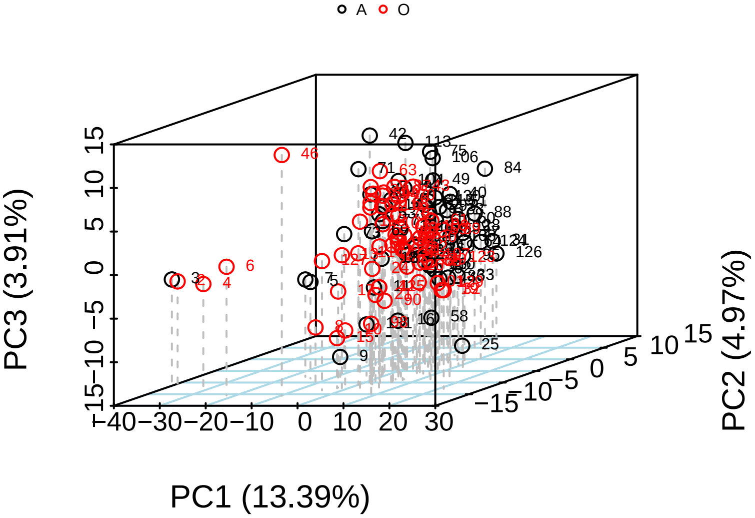
A
O
PC1 (13.39%)
PC2 (4.97%)
PC3 (3.91%)
−40 −30 −20 −10 0 10 20 30
−15 −10 −5 0 5 10 15
−15 −10 −5 0 5 10 15
